# Editorial: Advances in connectome-wide association studies (CWAS) along the neurodegeneration trajectory

**DOI:** 10.3389/fnagi.2023.1329854

**Published:** 2023-11-13

**Authors:** Chenfei Ye, Tao Wu, Andreia V. Faria

**Affiliations:** ^1^International Research Institute for Artificial Intelligence, Harbin Institute of Technology at Shenzhen, Shenzhen, China; ^2^Department of Neurology, Center for Movement Disorders, Beijing Tiantan Hospital, Capital Medical University, Beijing, China; ^3^China National Clinical Research Center for Neurological Diseases, Beijing, China; ^4^Department of Radiology, Johns Hopkins University School of Medicine, Baltimore, MD, United States

**Keywords:** neurodegeneration, disconnectome, Alzheimer's disease, brain network, CWAS

Brain structural and functional alterations have been consistently proposed to be involved in the neurobiological underpinnings of aging and neurodegenerative disorders, such as Alzheimer's disease (AD) and Parkinson's disease. Given that pathological perturbations of the central nervous system are often intertwined with brain connectivity alteration, it is becoming increasingly accepted that connectome reorganization plays a key role in determining cognitive or motor disability. The advances in connectome-wide association studies (CWAS) have the potential to allow for the identification of novel neural correlates of neurodegeneration at the whole-brain scale, elucidating how brain-network topology can shape neural responses to neurodegenerative damage.

This Research Topic aims to encourage the proposal of state-of-the-art methodologies related to CWAS techniques and their applications to neurodegenerative disorders. A total of five studies collected in this topic mainly cover (1) advanced statistical methods to avoid multiple covariates in analyzing brain connectivity; (2) a data-driven CWAS design to clarify and validate brain network findings associated with aging in a large sample of subjects; (3) a virtual analytical approach to describe brain disconnectome reorganization while avoiding the issue of artifacts that are prone to occur in the presence of white matter hyperintensity; (4) advanced dynamic connectivity measurements as novel brain imaging markers to discriminate AD patients at different stages; and (5) the brain structural covariance network analysis and related novel statistical model to unravel pathological progression of AD. In what follows, we summarized the highlights the methodologies and applications in aging and related neurodegeneration of each article.

Functional connectivity (FC) comparison across individuals can be challenging in CWAS due to complex covariates in inter-subject variations. Smith et al. proposed a subject-level regression model to consider multiple covariates, including parcel-wise geographic/homotopic distance and region or network belongings, to move away from reliance on localization assumptions underlying FC comparisons. The key point is to keep each individual's FC in its own geometry, without morphing to a common template. This could be extraordinarily useful when the extent and location of lesions to brain regions varies across subjects. Additionally, the authors demonstrated high repeatability of this model achieved in individual space with test-retest individual scans.

How aging affects functional networks of the brain in both within-network and between-network connectivity is unclear. To comprehensively explore this effect, Du et al. applied a priori-driven independent component analysis, named NeuroMark, on 6,300 healthy adult with an age range of 49–73 years from UK Biobank project. They highlighted multiple significant joint between-network FC changes related to aging occurred in default mode and sub-cortical networks. This spatial diversity of brain network reorganization revealed by CWAS may shed new light on mechanism underlying aging-related brain changes.

Another study conducted by Li et al. addressed the focal effect when analyzing brain structural network. To avoid potential confounds of white matter hyperintensity (WMH) in CWAS, the authors proposed a virtual lesion approach to estimate brain connectivity changes in aging. Specifically, WMH frequency maps across age ranges (50's, 60's, 70's, and 80's) were used to generate virtual lesion masks for each decade as regions of avoidance in white matter tractography. They quantitatively described the spatial disconnectome evolving patterns in cortical and subcortical areas, which might underlie cognitive and sensorimotor deficits seen in aging. The advancement of this virtual lesion approach can further help identify brain disconnectome features contributing to dementia risk.

Emerging evidence has supported that abnormal brain structural or functional connectivity could have been observed 10–20 years prior to the onset of clinical symptoms of AD. Aiming to clarify inconsistent brain FC findings in early stage of AD, the study conducted by Penalba-Sánchez et al. compared multiple FC measurements (i.e., static FC, dynamic FC using Pearson's correlation, sliding-windows correlation analysis, and the point process analysis) and evaluated their corresponding network segregation and integration in mild cognitive impairment and AD. The authors unexpectedly observed an increase in late stage AD and a slight decrease of FC in early MCI, and explained the potential underlying cognitive mechanism. Understanding the dynamic and non-linear nature of FC might be crucial for unraveling the unclear neuropathological factors affecting behavior symptoms along the trajectory of AD.

Unlike structural connectivity, which is classically represented by the connection strength of white matter tracts on individual level, brain structural covariance network (SCN) analysis highlights synchronized gray matter atrophy undergoing neurological pathological processes across brain regions on population level. Xiao et al. applied SCN analysis using T1-weighted MR images from a longitudinal cohort to examine whether and how earlier brain atrophy patterns impact the progressive changes of gray matter atrophy in AD patients over time. After establishing regular structural connectivity by Pearson correlation and causal structural connectivity by Granger causal analysis, they used a new approach named gray matter based spatial statistics to measure the gray matter volume at the core of the cortical plate, aiming to alleviate partial volume effect and inter-subject variability. High discrimination accuracy based on SCN connectivity features suggests that SCN approach may help identify the unique pathological progression of AD and other types of neurodegeneration.

In sum, the collective findings represented in these articles reflect the rapidly expanding development of the CWAS field. One could further expect a rising body of brain network analytical tools and statistical models updated in this emerging area, providing new avenues for identification of brain network markers related to aging and neurodegenerative disorders. However, whether and to what degree the brain network construction procedures (i.e., brain atlas selection, linear or non-linear connectivity definition) affects robustness of findings in neurodegeneration is still in dispute. Therefore, before further translating into clinical applications such as individual auxiliary diagnosis and treatment decision-making, we must be cautious whether the reliability and validity performance of these CWAS approaches has been fully reported and validated in large-sample and multi-center brain imaging data.

## Author contributions

CY: Writing—original draft. TW: Writing—review & editing. AF: Writing—review & editing.

